# Experimental Investigation of the directional collapse and microjet dynamics of single acoustic bubbles in confined tubes

**DOI:** 10.1016/j.ultsonch.2026.107841

**Published:** 2026-04-01

**Authors:** Hao Wu, Teng Zhang, Yongcheng Fang, Ruixiang Yu, Yongzhen Jin, Claus-Dieter Ohl, Yuanyuan Li

**Affiliations:** a Innovative Institute of Chinese Medicine and Pharmacy, Shandong University of Traditional Chinese Medicine, Jinan 250355, PR China; b Department of Soft Matter, Institute of Physics, Otto-von-Guericke University Magdeburg, Magdeburg 39106, Germany; c School of Medical Informational Engineering, Shandong University of Traditional Chinese Medicine, Jinan 250355, PR China

**Keywords:** Acoustic cavitation, Confined geometry, Boundary effects, Single acoustic cavitation bubble, Microjet dynamics

## Abstract

Understanding the behavior of single cavitation bubbles under ultrasonic excitation is crucial for biomedical applications, particularly targeted drug delivery. This study experimentally investigates the physics of acoustically driven bubble shape instabilities within confined tubes (inner diameters 600–800 μm) designed to mimic human micro-vessels. Utilizing an ultrafast photomicrographic system, we captured high-resolution recordings of the spatiotemporal evolution and translational trajectories of micrometric air bubbles. The experimental results demonstrate that when a bubble is positioned on the tube’s axial line, an increase in acoustic pressure significantly alters bubble morphology and reduces collapse time. Notably, the formation of high-velocity upward microjets was observed during collapse phases, with peak jet velocities scaling positively with ultrasonic intensity. A comparative analysis of varying initial bubble sizes reveals that the tube boundary induces a fourth-harmonic acoustic response, which substantially enhances the intensity of the bubble’s horizontal migration, rotational dynamics, and collapse violence. Furthermore, the initial proximity of the bubble to the tube wall was identified as a critical determinant of cavitation dynamics. Detailed analysis shows that a shorter initial distance to the boundary accelerates the rotational path of the bubble towards the wall, leading to an earlier and more intense asymmetric collapse. These findings provide new quantitative insights into the microscopic mechanisms of ultrasound-induced cavitation in confined geometries, offering a framework for optimizing ultrasonic applications in clinical and microfluidic environments.

## Highlights

• Cavitation dynamics were characterized in 600–800 μm bio-mimetic tubes.
• Acoustic pressure and bubble size dictate the microjet velocity and direction.
• Harmonic resonance was found to enhance bubble–boundary interactions in tubes.
• Critical stand-off distances trigger the transition to violent fragmentation.
• Microjet orientation rotates from axial to wall-normal based on wall proximity.

## 1 Introduction

In recent years, ultrasonic cavitation has attracted increasing attention from researchers due to its simple excitation method and the intense physical effects it can generate, leading to a wide range of applications in biomedicine [1], food processing [2], and industrial cleaning [3]. As research in this field has progressed, most existing investigations into the mechanisms and effects of ultrasonic cavitation have primarily relied on experiments employing materials with different properties under diverse geometrical configurations and environmental conditions. Such approaches aim to elucidate the variations in cavitation behavior and its associated effects across different scenarios [4], [5].

To date, studies in this area have predominantly focused on structural configurations to simulate boundary conditions relevant to various application scenarios, thereby clarifying the interaction mechanisms between cavitation bubbles and complex geometries [6], [7]. Typical structural shapes include planar surfaces, curved surfaces, microchannels, microporous structures, cavity structures, as well as various coupled configurations derived from these basic geometries. Investigations under these structural conditions are associated with a wide range of practical applications and significant engineering relevance [8]; however, they do not necessarily capture all aspects of cavitation behavior under strong geometric confinement. For instance, studies on cavitation bubble collapse–induced material damage based on planar structures have demonstrated, through experimental and numerical analyses, that microjet impacts and pressure waves generated during bubble collapse near a solid planar surface are the primary mechanisms responsible for mechanical damage and fatigue erosion of material surfaces. This effect has been extensively applied to the evaluation of cavitation resistance in underwater engineering materials such as propellers [9]. Moreover, in microchannel structures, ultrasonic cavitation can induce complex coupled interactions between bubbles and the surrounding fluid within microfluidic channels, resulting in the detachment of bubbles or particles adhered to the channel walls. This phenomenon provides experimental evidence for the acoustic removal of contaminants in microchannels [10]. In biomedical applications, ultrasonic cavitation has also been shown to enhance mass transfer across membrane systems. At both the membrane filtration system level and the cellular membrane scale, cavitation and acoustic perturbations can significantly improve transmembrane transport efficiency, thereby offering theoretical and experimental support for applications in drug delivery [11], [12].

Driven by the expanding scope of ultrasonic cavitation in biomedical engineering, systematic investigations have addressed the boundary dynamics and interfacial interactions between cavitation bubbles and microtube walls. Research to date has focused primarily on the mechanical responses and damage evolution of microtubes with varying material compositions under cavitation loading [13]. These studies indicate that rigid materials, owing to their high acoustic impedance and large elastic modulus, exhibit strong reflection of shock waves generated during cavitation collapse. As a result, localized transient pressures can reach the order of several gigapascals, inducing dense micro-pitting or flake-like spallation on the tube wall surface, and in severe cases, even leading to perforation of the microtube wall [14], [15].In contrast, elastic materials display fundamentally different energy dissipation mechanisms. Their compliant tube walls undergo localized indentation at the moment of bubble collapse, absorbing a portion of the impact energy through structural deformation and thereby effectively reducing peak pressure. However, under repeated cavitation loading, such materials may experience irreversible plastic deformation or surface wrinkling, which can ultimately compromise the geometric stability and fluid sealing performance of the microtubes [16], [17]. Further studies have revealed that fatigue effects become particularly pronounced under long-term ultrasonic exposure [18]. Polymer microtubes exhibit a reduction in Young’s modulus due to chain scission and degradation of cross-linked networks, whereas metal microtubes tend to develop microcracks in regions of stress concentration, which may ultimately lead to fatigue fracture [19]. Most of these investigations adopt an integrated multimodal characterization strategy, combining high-speed imaging to capture the transient interactions between bubbles and tube walls, surface morphology analysis to track damage evolution, quantitative mechanical property testing, and full-field strain reconstruction of tube walls achieved by coupling fluorescent microsphere tracking with digital image correlation techniques. This comprehensive approach enables the establishment of a coherent understanding spanning from microscopic cavitation dynamics to macroscopic material failure mechanisms [20].

Although single bubbles in microtube structures have been investigated regarding collapse asymmetry, jet formation, and pressure wave propagation, research focused on specific microtube configurations remains limited. In existing studies, cavitation bubbles are typically fully confined during expansion, resulting in prolate ellipsoidal morphologies[21]. However, such high confinement does not represent the diversity of biomedical scenarios, such as drug delivery in large blood vessels where bubble dimensions are small relative to the vessel diameter. Furthermore, most contemporary studies utilize laser-induced cavitation or spark discharge methods; while useful, these techniques do not fully replicate the physical reality of acoustically driven bubbles. In industrial contexts—such as the cleaning of cooling microchannels, fuel nozzles, and microreactor conduits—the structures are essentially cylindrical, yet the bubbles remain significantly smaller than the tube diameter[22], [23]. In these cases, ultrasound is the primary driver of cavitation. Consequently, investigating cavitation within microtubes under an ultrasonic field allows for a more accurate recapitulation of bubble–wall interactions, enabling better prediction of cleaning efficiency and the risk of structural damage.

In this study, an experimental platform was developed to generate precisely controlled, micrometre-scale single bubbles within transparent tubes, while characterizing their dynamics in an ultrasonic field via high-speed photography. By systematically varying the acoustic pressure, initial bubble radius, and spatial positioning within the tube, distinct variations in bubble dynamics—including morphological transitions, microjet velocities, collapse temporalities, and rotational behaviours—were investigated. Several novel phenomena were identified through this experimental approach. The objective of this paper is to analyse the critical factors influencing the efficiency of acoustic cavitation within confined geometries, providing insights relevant to targeted drug delivery and the ultrasonic cleaning of industrial tubular structures.

## 2 Experimental methods

**Fig. 1:**
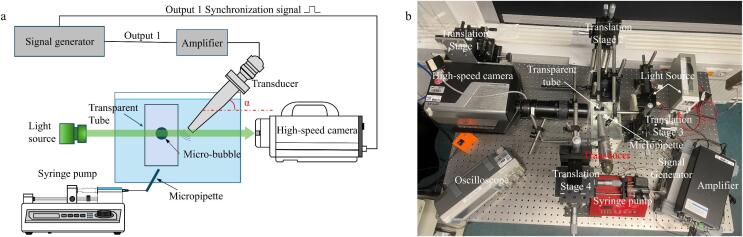
(a) Schematic of the experimental setup. (b) Photo of the experimental setup.

In clinical settings, ultrasound-induced cavitation bubbles are generated during the rarefaction phases of acoustic waves and typically manifest as cavitation clusters within the vasculature[24]. To elucidate the spatiotemporal evolution of these bubbles, individual air bubbles were generated utilizing a syringe-coupled micropipette assembly[25] High-resolution dynamics were captured using a high-speed camera (Shimadzu X2 or Photron SA-Z, Japan) operating at a frame rate of 200 kfps. Acoustic excitation was provided by an ultrasonic horn transducer driven by a power amplifier (PiezoDriver PD200, Australia) at a frequency of 16.6 kHz, the ultrasonic transducer was aligned at an angle of approximately 30° (α) relative to the horizontal axis of the tube. The local acoustic peak pressure (*P*) was calibrated using a hydrophone (TC4034, Teledyne RESON) at the bubble nucleation site. The vessel models were fabricated with the sacrificial layer technology. PMMA (polymethyl methacrylate) was chosen as the sacrificial layer and PDMS (Polydimethylsiloxane) was chosen as the wall of tubes. The schematic diagram and the picture of experimental set-up are shown in Fig. 1a and Fig. 1b, respectively.

In this paper, the acoustic peak pressure (*P*) varies from 1.5 kPa to 6.7 kPa, the initial radius of air bubbles (*R*_0_) varies between 15–85 μm, the experimental liquid (clean deionized water) temperature was 22 ^◦^C, the inner diameter of the tube (*d*) varied from 0.6 mm to 0.8 mm and the length is 30 mm, and the wall thicknesses (*h*) used in experiments are 0.2 mm, 0.4 mm respectively. In this experiment, the bubbles in the free field were also induced.

## 3 Results and Discussion

### 3.1 Response of single bubbles on the tube axis under varying ultrasonic intensities

In this section, the influence of acoustic pressure on the dynamics of a single cavitation bubble—specifically its shape, the collapse dynamics, and the movement—was investigated for bubbles initially close to the center axis of the tube. Single air bubbles with an initial radius (*R*_0_) of 85 μm were generated at the center of a tube (inner diameter *d* = 800 μm, wall thickness *h* = 200 μm. Three different acoustic pressure amplitudes, *P_A_*, are studied, namely 1.5 kPa, 3.5 kPa, and 6.7 kPa, all operated at a frequency *f* = 16.6 kHz. The characteristic response of the gas bubbles to the acoustic field included amplitude oscillations, axial migration, and high-velocity collapse.

**Fig. 2:**
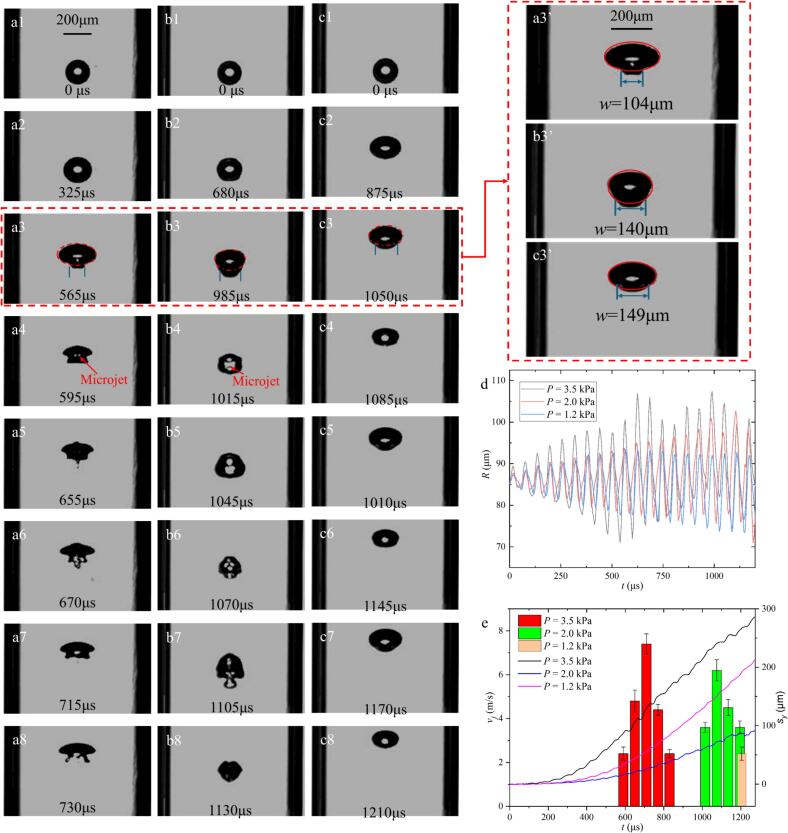
Comparison of the dynamic behaviour of bubbles in ultrasonic filed with different peak pressure *P*: (a) *P* = 6.7 kPa. (b) *P* = 3.5 kPa. (c) *P* = 1.5 kPa. with a frequency of 16.6 kHz. The initial radius of the bubble is *R*_0_ = 85 μm in the center of a tube with the inner diameter (*d*) of 800 μm and the thickness (*h*) of 200 μm. (d) Graph of bubble radius (*R*) vs. time (*t*). (e) Speed of microjets during bubbles collapse *v_j_* (n = 7, bar charts with three different colours) and upward vertical movement distance of the bubble *S_y_* versus *t* (line charts).

Fig. 2 illustrates the high-speed photographic sequences of bubble evolution along the axial line. The bubbles in absence of a sound field rise within the tube with a velocity of 16 μm/ms upwards. At time *t* = 0 we switch on the sound field. The first column in Fig. 2, presents the response of the bubble driven by an acoustic pressure amplitude of *P_A_* = 6.7 kPa (Fig. 2a), the bubble initially exhibits spherical oscillation. By approximately 325 μs, it transitions to an ellipsoidal morphology (Fig. 2a2), a possible explanation could be the increasing translational velocity and the inherent asymmetry of the acoustic field along the tube axis. Subsequently, the bubble evolves into a spherical sector shape by 570 μs (Fig. 2a3). As the acoustic pressure drives more intense deformation, the distal interface undergoes rapid involution, then resulting in an upward-directed microjet under gravity-induced asymmetry (Fig. 2a4). This jetting phenomenon is cyclical and occurs without immediate bubble fragmentation; the collapse intensity and jet velocity increase progressively across subsequent cycles (Fig. 2Figs. 2a5-6 and a7-8).

At a moderate pressure of *P_A_* = 3.5 kPa (Fig. 2b), the response of the bubble follows a similar trend, though the duration of spherical stability is extended to approximately 680 μs before ellipsoidal deformation occurs (Fig. 2b2). The axial migration speed is reduced, likely due to the near-cancellation of the acoustic radiation force and buoyancy. Following five cycles of axisymmetric oscillation, the bubble assumes a diamond-like shape (Fig. 2b3) and undergoes collapse with a corresponding microjet (Fig. 2b4). While cyclical jetting persists (Fig. 2Figs. 2b5–2b8), the bubble does not shrink as much as for *P_A_* = 6.7 kPa.

At the lowest pressure of *P_A_* = 1.5 kPa (Fig. 2c), the transition to an ellipsoidal shape is further delayed to *t* = 875 μs. The shrinkage occurs cyclically without the formation of discernible microjets, as the bubble alternates between spherical sector and spherical cap morphologies (Fig. 2Figs. 2c3–2c8). To quantify these morphological variations, the characteristic length (*w*) of the spherical cap was analyzed (Fig. 2Figs. 2a3,2b3 and 2c3). The results indicate that *w* increases as acoustic pressure decreases, confirming that bubble deformation before the primary collapse is strongly pressure-dependent.

The analysis of varying ultrasonic intensities on the evolution of a single bubble along the tube axis indicates that the synergistic effects of buoyancy and the acoustic radiation force drive axial migration, oscillation, and collapse. The resulting collapse morphology is highly sensitive to fluctuations in the acoustic pressure. To quantify these dynamics, the radial evolution curves (*R*-*t* curves, Fig. 2d), microjet velocities (*v_j_*), and temporal upward displacements (*S_y_*, Fig. 2e) were evaluated. As illustrated in Fig. 2d, both the maximum radius (*R_max_*) and the oscillation amplitude decrease as the peak acoustic pressure *P* is reduced from 6.7 kPa to 1.5 kPa, a trend consistent with previous literature [25].

Quantitative data in Fig. 2e reveals that the highest microjet velocity occurs during the third oscillation period at *P_A_* = 6.7 kPa and the second period at *P_A_* = 3.5 kPa, with velocities declining in subsequent cycles. Furthermore, higher acoustic pressures lead to an earlier collapse. Interestingly, the bubble exhibits its minimum axial displacement not for the smallest pressure but for *P_A_* = 3.5 kPa. This is likely due to the competition between buoyancy and the primary Bjerknes force within the traveling wave field. At *P_A_* = 6.7 kPa, the significantly larger oscillation amplitude enhances the pressure gradient between the upper and lower bubble poles, resulting in the highest observed migration velocity (Fig. 2e).

Further experiments were conducted to assess the influence of increased spatial confinement. While the internal acoustic field is affected by the tube dimensions and material properties, qualitative comparisons of the translational trajectories and morphological evolutions suggest that bubbles in narrower tubes are more susceptible to boundary-induced effects. In these cases, the bubble exhibits an increased propensity to deviate toward the tube wall, as documented in Supplementary Videos 1–3.

### 3.2 Effects of bubble radius on dynamics of single acoustic cavitation bubble on the tube axis

**Fig. 3:**
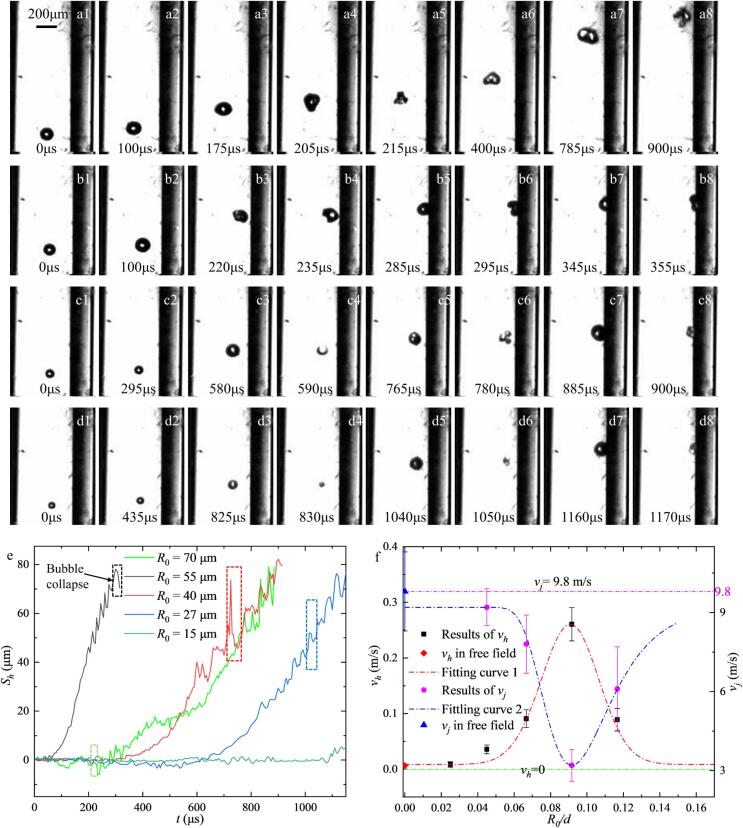
Comparison of the typical dynamic behaviours of single bubbles with different initial radii *R*_0_: (a) *R*_0_ = 70 μm. (b) *P* = 55 μm. (c) *P* = 40 μm. (d) *P* = 70 μm in the centre of a transparent tube (d = 600 μm, h = 400 μm) in an ultrasonic field (f = 16.6 kHz, P = 3.5 kPa). (e) Horizonal movement distance of the bubble toward the tube right boundary *S_y_* versus *t* for bubbles with various initial radii. (f) Speed of bubble’s horizonal movement *v_h_* vs. *R*_0_/*d* (black squares, n = 6), the red diamond shape represents the result of *v_h_* when bubbles are in free field; speed of microjets during bubbles collapse *v_j_* (magenta circles, n = 6), the blue triangle represents the result of *v_j_* when bubbles are in free field.

In this section, the influence of the initial bubble radius (*R*_0_) on the volume dynamics was investigated under constant acoustic conditions (*P_A_* = 3.5 kPa, *f* = 16.6 kHz) within a tube of increased confinement (*d* = 600 μm, *h* = 400 μm). Single bubbles with *R*_0_ ranging from 15 to 70 μm were analyzed. As shown in Fig. 3, the reduced tube diameter significantly alters the bubble response compared to the results in Section 3.1. During the initial acoustic cycles, all bubbles, regardless of size, manifest in a purely oscillatory regime while maintaining a spherical morphology and migrating axially (Fig. 3Figs. 3a–d1–2).

As the temporal evolution proceeds, larger bubbles (*R*_0_ = 70 μm) transition to an ellipsoidal morphology (Fig. 3a3) and undergo an earlier formation of a jet at 205 μs. Then an axisymmetric upward microjet occurs (Fig. 3Figs. 3a4–5), followed by a lateral migration toward the right tube boundary—consistent with the direction of ultrasonic propagation. As the bubble approaches the wall, the microjet orientation rotates, aligning with the boundary proximity (Fig. 3Figs. 3a6–8). Conversely, smaller bubbles exhibit slower lateral migration and irregular oscillations (Fig. 3Figs. 3b2–4, c2–4, d2–4), driven by nonlinear acoustic radiation forces. For these smaller bubbles, once the distance to the wall (*l*) reaches a critical threshold, they develop shape modes and collapse with microjets directed toward the boundary at an oblique angle (Fig. 3Figs. 3b–d5–6). Notably, the collapse time is delayed with decreasing *R*_0_.

The horizontal bubble position as a function of time for several *R*_0_ is presented in Fig. 3e. Bubbles with *R*_0_ = 55 μm exhibit the highest velocity toward the right boundary and manifest distinct post-collapse rebounding (highlighted in the boxes of Fig. 3e). The average horizontal velocity (*v_h_*) and the initial microjet velocity (*v_j_*) are plotted in Fig. 3f as a function of the dimensionless ratio *R*_0_/*d*. Baseline experiments in an unconfined free field (Supplementary Videos 4–9) yielded near-zero horizontal velocities, suggesting that the “lateral oscillation” observed in the traveling wave field is a localized instability because of the complex ultrasonic field induced by the tube structure. Furthermore, while the 55 μm bubble migrates and collapses most rapidly, its microjet velocity is the lowest among the groups studied. This is attributed to insufficient energy accumulation during its shortened expansion phase, a phenomenon consistent with prior observations in confined cavitation dynamics [26].

Furthermore, our observations of the bubble dynamics that when low-frequency ultrasound is emitted from the upper-right direction relative to the horizonal line, the direct impact of the traveling acoustic waves on the bubble is relatively negligible, given that the bubble radius is significantly smaller than the acoustic wavelength. This is consistent with prior findings in free-field bubble research[27]. Instead, the bubble's lateral migration is primarily governed by complex reflected waves from the tube walls, and the magnitude of this effect is intrinsically linked to the bubble size. As shown in Fig. 2, larger bubbles remain relatively unaffected by acoustic transmission and reflection, maintaining a near-zero horizontal displacement. Conversely, for smaller bubbles, the lateral migration speed decreases as the initial radius (*R*_0_) is reduced. Notably, bubbles with dimensions closer to the higher-order harmonic resonance radius exhibit the most pronounced rightward migration. This underscores the critical role of the acoustic field frequency in driving horizontal or radial motion; the ultrasound propagating toward the lower-right appears only to delay the wall-ward migration of smaller bubbles. It can be inferred that as the angle between the transducer axis and the horizontal plane decreases, the influence of the transverse harmonic resonant waves intensifies. Consequently, bubbles of a given size would migrate toward the wall at higher velocities. Bubbles with radii approaching the resonant dimensions of the acoustic field will not only exhibit faster migration but also undergo the most violent collapse.

**Fig. 4:**
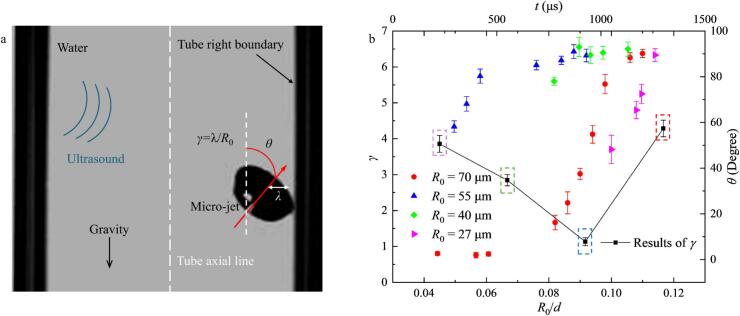
(a) Schematic description for the collapse position parameter *γ* and the angle *θ.* (b) The results of the collapse position parameter *γ* vs. *R*_0_/*d* (black squares, n = 6) and the angle *θ* value vs. time (*t*) for bubbles with different initial radii (red circles for *R*_0_ = 70 μm, blue triangles for *R*_0_ = 55 μm, green diamond shapes for *R*_0_ = 40 μm and magenta oblique triangles for *R*_0_ = 27 μm).

To further characterize the mechanisms of bubble collapse near the boundary, a dimensionless distance parameter, *γ =* λ/*R*_0_, was defined, where λ represents the distance between the bubble center and the right tube boundary (Fig. 4a). Additionally, the angle *θ* between the microjet direction (the axis of bubble symmetry) and the tube center line measures the orientation during collapse to understand the competing influence between the boundary and buoyancy. The angle *θ* and the distance *γ* are plotted for bubbles with initial radii of 27, 40, 55, and 70 μm in Fig. 4b. To illustrate the correlation between *γ* and *θ*, color-coded dashed boxes (red, blue, green, and rose) were employed to represent specific *γ* values corresponding to each *R*_0_.

The results indicate that *γ* reaches its minimum value when *R*_0_ = 55 μm, suggesting that bubbles of this size exhibit the most vigorous translational and collapse dynamics. This intensified response is likely due to fourth-harmonic resonance; the resonant radius calculated via the Minnaert equation for this ultrasonic field is approximately 55 μm (the resonance frequency of a 55 μm bubble is approximately 66.4 kHz according to the Minnaert equation). Furthermore, while the microjet angle *θ* eventually converges to approximately 90° for all bubbles − indicating normal impingement upon the tube wall − the 55 μm bubbles reach this orientation at the highest rate. These findings demonstrate that bubble size significantly dictates the efficiency of bubble–boundary interactions within a specific acoustic field. Consequently, selecting an optimal bubble size is critical for enhancing the efficacy of ultrasonic applications in confined geometries, such as industrial tube cleaning or targeted clinical vessel therapies.

### 3.3 Initial spatial positioning on bubble dynamics in a confined ultrasonic field

Building on the results from Section 3.2, which indicated that bubble collapse is triggered by boundary proximity, this section systematically investigates the relationship between the bubble’s initial position (*l*) and its dynamic evolution. Single bubbles (*R*_0_ = 75 μm) were generated at varying normalized stand-off distances (*l/d*) of 0.5, 0.42, 0.34, 0.25, 0.16, and 0.08 within a tube (*d* = 800 μm, *h* = 200 μm). A constant low-intensity acoustic pressure (*P* = 1.5 kPa, *f* = 16.6 kHz) was applied to isolate the boundary-induced effects on microjet generation.

**Fig. 5:**
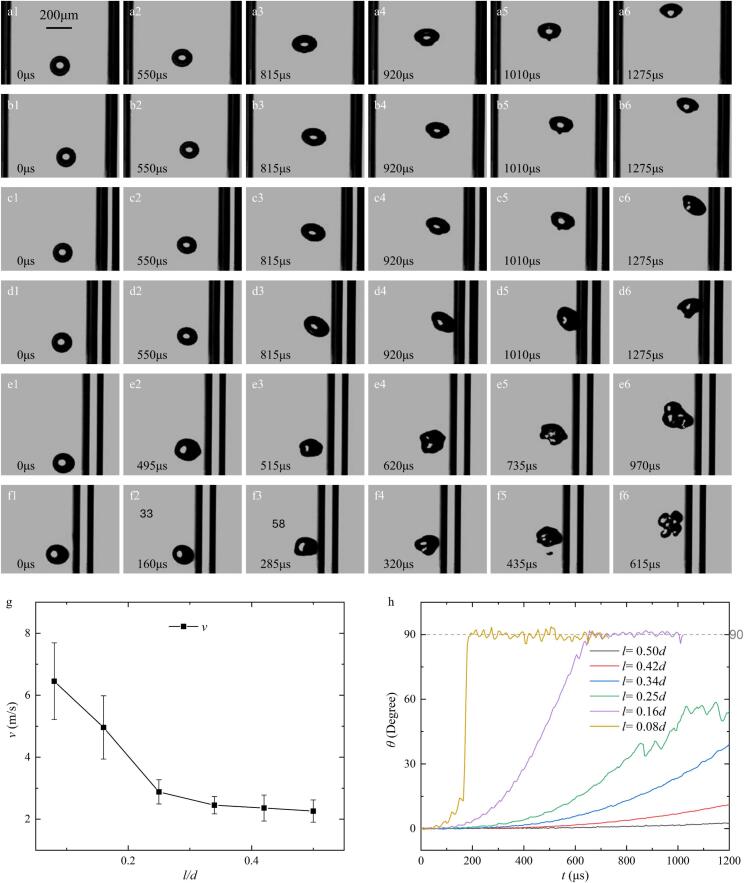
Comparison of the dynamic behaviour of bubbles (*R*_0_ = 75 μm) in different positions inside the tube (*d* = 800 μm, *h* = 200 μm) in an ultrasonic field (*P* = 1.5 kPa, *f* = 16.6 kHz): (a) *l/d* = 0.5. (b) *l/d* = 0.42. (c) *l/d* = 0.34. (d) *l/d* = 0.25. (e) *l/d* = 0.16. (f) *l/d* = 0.08. (g) Velocities of microjets (or the fastest speed of the bubble margin if no microjet occurs) vs. the value of *l/d* (n = 7); (h) The typical angle (*θ*) vs. time *t* for bubbles with various initial value of *l/d*.

As shown in Fig. 5a, bubbles initially positioned on the tube axis (*l* = 0.5*d*) exhibit symmetric oscillation and morphological transitions consistent with the observations in Section 3.1. However, as the initial distance decreases (e.g., *l* = 0.42*d*), the bubble initially rises spherically but gradually develops an ellipsoidal morphology with a symmetrical axis that tilts toward the proximal right boundary (Fig. 5b2). As the bubble continues to migrate laterally, the inclination angle (*θ*) increases (Fig. 5g). For bubbles generated at *l* = 0.34*d* and 0.25*d* (Fig. 5Figs. 5c and 5d), the lateral migration velocity is markedly higher, and the rate of angular rotation (*θ*) accelerates. Notably, at *l* = 0.25*d*, the lower interface undergoes concave involution (Fig. 5d6), signaling the onset of asymmetric collapse even under low-intensity irradiation.

A critical proximity threshold is observed at *l* = 0.16*d*. In this regime, the bubble oscillates near the boundary until approximately 495 μs (Fig. 5Figs. 5e1–2) before undergoing a stable collapse characterized by a concave deformation on its distal side (Fig. 5e3). This transitions into cyclical jetting with increasing intensity over time, eventually culminating in a high-velocity microjet impingement on the wall (Fig. 5e6). At the most extreme proximity (*l* = 0.08*d*, Fig. 5f), the bubble undergoes rapid compression-expansion cycles. It reaches its maximum volume at 285 μs (Fig. 5f3) and immediately collapses with high intensity—significantly earlier than bubbles at larger *l*. This violent collapse results in high-speed microjets that induce droplet “pinching-off” (Fig. 5f5) and catastrophic bubble fragmentation, resulting in a characteristic flower-like morphology (Fig. 5f6). These results demonstrate that initial boundary proximity is a decisive factor in determining the collapse temporality, rotational velocity, and the transition from stable oscillation to violent jet-driven fragmentation.

To quantify the collapse intensity across varying initial stand-off distances (*l*), the microjet velocities (*v*) were calculated and compared (Fig. 5g). For cases where the bubble did not reach a violent collapse threshold within the observation window or period, the maximum interfacial velocity was utilized as a proxy for “microjet speed” to characterize the movement intensity. Additionally, the temporal evolution of the angle *θ*—representing the orientation of the bubble's symmetry axis relative to the tube’s axial line—was recorded (Fig. 5h).

As illustrated in Fig. 5g, the mean peak velocity of the bubble interface during the primary collapse decreases from 6.45 ± 1.24 m/s to 4.96 ± 1.02 m/s as the normalized distance *l/d* increases from 0.08 to 0.16. For bubbles exhibiting stable oscillations without collapse, the maximum interfacial velocity similarly declined from 2.88 ± 0.39 m/s to 2.26 ± 0.36 m/s as *l/d* increased from 0.25 to 0.5. These results demonstrate that the initial proximity to the tube boundary is a critical determinant of cavitation dynamics in low-frequency ultrasonic fields, directly influencing both the collapse temporality and the resulting intensity.

Furthermore, the orientation angle *θ* effectively tracks the reorientation of the bubble from a buoyancy-driven upward direction to a boundary-driven lateral direction. The rate of this angular increase was found to escalate significantly as *l* decreased from 0.50d to 0.08d. This acceleration confirms that a closer initial proximity to the boundary enhances the efficiency of the interfacial interaction and momentum transfer between the bubble and the tube wall. Based on the experimental setting, the dynamic evolution of the bubbles in these confined microtubes is further influenced by secondary acoustic effects, notably microstreaming and rectified diffusion. As the bubble oscillates at 16.6 kHz, the resulting acoustic microstreaming generates localized secondary flows. In a tube, these flows are constrained by the walls, potentially enhancing the pressure asymmetry that leads to the observed microjet rotation. Furthermore, the stability of the bubble over multiple acoustic cycles-prior to the violent collapses shown in Fig. 5-is supported by rectified diffusion. This mechanism ensures that the gas concentration within the bubble remains sufficient to sustain nonlinear oscillations despite the high internal pressures reached during the compression phases.

## 4 Conclusions

In this study, the dynamic behavior of single acoustic cavitation bubbles within a confined tube environment was systematically investigated using high-speed photography. By examining the influences of ultrasonic intensity, initial bubble radius, and proximity to the tube boundary, the following conclusions are drawn:

Impact of Ultrasonic Intensity: The intensity of the ultrasonic field significantly dictates the bubble's oscillation mode and collapse severity. Higher peak pressures (6.7 kPa) lead to larger maximum radii, greater vibration amplitudes, and earlier collapse times. While bubbles remain primarily on the tube’s axial line under these conditions, the transition from stable spherical oscillation to violent collapse with upward microjets is accelerated by increased acoustic power.

Role of Resonance and Bubble Size: The interaction between the bubble and the tube boundary is highly sensitive to the initial bubble radius (*R*_0_). Bubbles with a radius near the resonant size (approximately 59 μm for the fourth harmonic of the 16.6 kHz field) exhibit the most intense dynamics, including the fastest horizontal migration toward the tube wall and the earliest rotation of the collapse axis toward the boundary. This suggests that tuning bubble size is critical for optimizing applications like ultrasonic cleaning within narrow tubes and drug delivery through vessels.

Boundary Proximity and Jetting Direction: The tube boundary acts as a primary driver for asymmetrical collapse. As the initial distance *l* between the bubble and the wall decreases, the bubble's symmetry axis rotates from the upward (buoyancy-driven) direction toward the wall-normal direction. A critical proximity threshold exists (approximately *l* < 0.16*d*) where the boundary influence overrides other forces, leading to high-speed microjets, bubble disruption, and the formation of “flower-shaped” fragments upon collapse.

Coupled Force Dynamics: The trajectory and morphology of the bubble are governed by a complex competition between buoyancy, acoustic traveling wave forces, and boundary-induced Bjerknes forces. In confined geometries, these forces determine the “horizontal wiggle” and the specific angle (*θ*) of microjet impact, which eventually reaches 90° (vertical to the wall) as the bubble nears the boundary.

These findings provide a quantitative basis for controlling cavitation activity in confined liquid-filled tubes. Understanding these thresholds for microjet formation and directional rotation is essential for advancing ultrasonic medical therapies and microfluidic cleaning technologies.

## CRediT authorship contribution statement

**Hao Wu:** Writing – review & editing, Writing – original draft, Visualization, Validation, Software, Resources, Project administration, Methodology, Investigation, Funding acquisition, Formal analysis, Data curation, Conceptualization. **Teng Zhang:** Writing – original draft, Visualization, Investigation. **Yongcheng Fang:** Validation, Resources. **Ruixiang Yu:** Methodology, Formal analysis. **Yongzhen Jin:** Resources, Methodology, Investigation. **Claus-Dieter Ohl:** Writing – review & editing, Supervision. **Yuanyuan Li:** Supervision, Conceptualization.

## Declaration of competing interest

The authors declare that they have no known competing financial interests or personal relationships that could have appeared to influence the work reported in this paper.
